# Increased Cesarean Section Rates during the COVID-19 Pandemic: Looking for Reasons through the Robson Ten Group Classification System

**DOI:** 10.1055/s-0043-1772182

**Published:** 2023-08-18

**Authors:** Cassia Elane Berbel da Silva, Jose Paulo Siqueira Guida, Maria Laura Costa

**Affiliations:** 1 Departament of Gynecology and Obstetrics, Faculdade de Ciências Médicas, Universidade Estadual de Campinas, Campinas, SP, Brazil

**Keywords:** cesarean section rates, COVID-19, Robson classification, taxas de cesárea, COVID-19, classificação de Robson

## Abstract

**Objective** To compare cesarean section (CS) rates according to the Robson Ten Group Classification System (RTGCS) and its indications in pregnant women admitted for childbirth during the first wave of the coronavirus disease 2019 (COVID-19) pandemic with those of the previous year.

**Materials and Methods** We conducted a cross-sectional study to compare women admitted for childbirth from April to October 2019 (before the pandemic) and from March to September 2020 (during the pandemic). The CSs and their indications were classified on admission according to the RTGCS, and we also collected data on the route of delivery (vaginal or CS). Both periods were compared using the Chi-squared (χ ^2^ ) test or the Fisher exact test.

**Results** In total, 2,493 women were included, 1,291 in the prepandemic and 1,202 in the pandemic period. There was a a significant increase in the CS rate (from 39.66% to 44.01%; *p* = 0.028), mostly due to maternal request (from 9.58% to 25.38%; *p* < 0.01). Overall, groups 5 and 2 contributed the most to the CS rates. The rates decreased among group 1 and increased among group 2 during the pandemic, with no changes in group 10.

**Conclusion** There was an apparent change in the RTGSC comparing both periods, with a significant increase in CS rates, mainly by maternal request, most likely because of changes during the pandemic and uncertainties and fear concerning COVID-19.

## Resumo

**Objetivo** Comparar as taxas de cesárea segundo a Classificação de Robson, assim como suas indicações, em mulheres admitidas para parto durante a primeira onda de doença do coronavírus 2019 ( *coronavirus disease 2019* , COVID-19, em inglês), com as do ano anterior.

**Materiais e Métodos** Conduzimos um estudo transversal que comparou as mulheres admitidas para parto entre abril e outubro de 2019 (pré-pandemia) e entre março e setembro de 2020 (durante a pandemia). As cesarianas e as suas indicações foram classificadas conforme o sistema proposto por Robson, e obteve-se a via de parto (vaginal ou cesárea). Ambos os períodos foram comparados usando-se os testes do Qui quadrado ou o exato de Fisher.

**Resultados** Ao todo, 2.943 mulheres foram incluídas, das quais 1.291 antes da pandemia e 1.202 durante a pandemia. A taxa de cesárea aumentou significativamente (de 39.66% para 44,01%; *p* = 0,028), principalmente devido a desejo materno (de 9,58% para 25,38%; *p* < 0,01). Os grupos 5 e 2 foram os que mais contribuíram para as taxas de cesárea. Durante a pandemia, o grupo 1 reduziu sua frequência, enquanto o grupo 2 a aumentou.

**Conclusão** Houve uma aparente mudança nas características da população conforme a classificação de Robson. Observou-se significativo aumento nas taxas de cesárea, principalmente por desejo materno, o que reflete possíveis incertezas e medos relacionados à COVID-19.

## Introduction

In 2020, the world faced a new and challenging situation: the coronavirus disease 2019 (COVID-19) pandemic, as declared by the World Health Organization (WHO) on March, 1 with significant consequences and increased maternal mortality rates. 2 Brazil, a country with heterogeneous social and economic conditions, was one of the most affected in terms of the rates of infections and mortality among pregnant women. 3

During pregnancy and the postpartum period, COVID-19 is associated with a high risk of developing severe acute respiratory syndrome (SARS); however, it also affects maternal anxiety due to the fear of complications during pregnancy and childbirth. 4 In addition, companions have experienced restrictions in the delivery rooms, leaving pregnant women with less support during delivery. 5 Regardless of the risks and fear, COVID-19 infection, especially in mild cases, is not an indication for cesarean delivery. 5 6 7

The WHO states that “there is no justification for any region to have a cesarean rate higher than 10-15%.” 8 However, there has been a progressive increase in cesarean rates around the world. 9 In 2016, the WHO, concerned about the global increase in cesarean rates and the negative consequences on maternal and child health, recommended the implementation of a universal classification to compare cesarean rates in different hospitals, cities or regions, and even within the same place, in different time frames, and proposed the use of a 10 group classification, known as the Robson Ten Group Classification System (RTGCS), which has been previously validated. 9 10

In Brazil, cesarean section (CS) rates (CSRs) have significantly increased in recent decades, at an alarming pace, from 38% in 1994 to 50% in 2009, reaching 57% in 2018. 11 12 There is a concern that the COVID-19 pandemic may have increased even more such rates.

The present study intends to evaluate the impact of the COVID-19 pandemic on the CSRs at a secondary public hospital in Southeastern Brazil. We also aimed to compare the pandemic period to the previous year, as well as to describe the contribution of each Robson group and the main indications for CS in the two periods.

## Materials and Methods

The present was a retrospective, cross-sectional study in which we reviewed the medical charts of all women admitted for childbirth at Hospital Estadual Sumaré (HES) from March to September 2020 (during the COVID-19 pandemic) and from April to October 2019 (before the pandemic). We determined the overall CSRs in both periods and the ten groups of the RTGCS, which considers the following obstetric characteristics: parity, previous CS, gestational age, onset of labor, fetal presentation, and the number of fetuses. These characteristics are mutually exclusive, fully inclusive, and clinically relevant, leading to a simple classification and enabling comparisons over time within a unit and among different units ( Chart 1 ).

**Chart 1:** Robson Ten Group Classification System ^10^

Group	Description
1	Nulliparous, singleton, cephalic, ≥ 37 weeks of gestation, in spontaneous labor
2	Nulliparous, singleton, cephalic, ≥3 7 weeks of gestation, induced labor or cesarean section before labor
3	Multiparous (excluding previous cesarean section), singleton, cephalic, ≥ 37 weeks of gestation, in spontaneous labor
4	Multiparous without a previous uterine scar, with singleton, cephalic pregnancy, ≥ 37 weeks of gestation, induced or cesarean section before labor
5	Previous cesarean section, singleton, cephalic, ≥ 37 weeks of gestation
6	All nulliparous with a single breech
7	All multiparous with a single breech (including previous cesarean section)
8	All multiple pregnancies (including previous cesarean section)
9	All women with a single pregnancy in transverse or oblique lie (including those with previous cesarean section)
10	All singleton, cephalic, < 37 weeks of gestation (including previous cesarean section)

Data were inserted into a Microsoft Office 2019 Excel (Microsoft Corp., Redmond, WA, United States) spreadsheet and analyzed with the Epi Info (Centers for Disease Control and Prevention, Atlanta, GA, United States) software, version 7.2.5. We initially considered the overall CSR in both periods, determined and compared the frequency of all RTGCS groups, the contribution of each group to the CSRs, and the indication for the CS. The results using the RTGCS were presented as recommended by the WHO. 12

To estimate the sample size, we considered that the population covered by the maternity hospital was of 286 thousand inhabitants, and supposed that CSR would be of around 40%. For a confidence interval (CI) of 99.99%, the minimum number of included cases would be 1,413.

Differences between groups and over the considered periods were presented using the Chi-squared (χ ^2^ ) test to determine the statistical significance of the data collected during the two periods, with values of *p* < 0.05 considered significant. We also obtained prevalence ratio (PR) and CIs to compare the two periods.

As the present is a retrospective study, without any clinical intervention, with the review of medical records and also considering that there is no postpartum follow-up at the institution, the informed consent form was waived by the local ethics committee, which approved the research protocol under CAAE 26439119.3.0000.5404. All the principles defined in the Declaration of Helsinki and in Resolution no. 466/12 of the Brazilian National Health Council were respected.

## Results

**Fig. 1:**
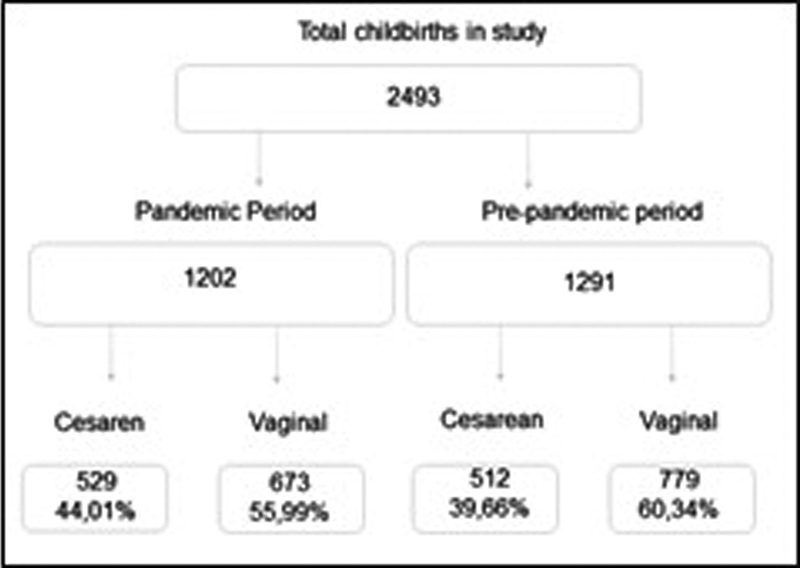
Flowchart of the women included in the present study.

A total of 2,493 deliveries were considered, 1,202 during the pandemic and 1,291 before it ( Fig. 1 ). Table 1 presents the overall distribution of deliveries and CSs during both periods. Group 3 was the most frequent in the sample, with 547 deliveries, followed by groups 5 (546 deliveries) and 1 (474 deliveries). There were 244 (9.79%) preterm deliveries during the period.

**Table 1:** Overall distribution of deliveries and cesarean sections (CSs) in a Brazilian maternity during a two-year period

Group	Number of CSs	Number of women	Percentage of women	CS rate (%)	Absolute group contribution to overall CS rate	Relative contribution of group to overall CS rate
1	90	474	19.01%	18.99%	3.61%	8.65%
2	221	339	13.60%	65.19%	8.86%	21.23%
3	32	547	21.94%	5.85%	1.28%	3.07%
4	69	219	8.78%	31.51%	2.77%	6.63%
5	382	546	21.90%	69.96%	15.32%	36.70%
6	23	23	0.92%	100.00%	0.92%	2.21%
7	48	51	2.05%	94.12%	1.93%	4.61%
8	41	47	1.89%	87.23%	1.64%	3.94%
9	3	3	0.12%	100.00%	0.12%	0.29%
10	132	244	9.79%	54.10%	5.29%	12.68%
Total	1.041	2.493	100.00%	41.76%		100.00%

The overall CSR was of 41.76% ( *n* = 1,041). Groups 5, 2 and 10 were the most important contributors to the rate ( Table 1 ).

Table 2 shows that there were important differences regarding the frequency of each RTGCS in the two periods evaluated. The prevalence among groups 2 and 5 increased during the pandemic, from 12.01% to 15.31% (PR: 1.275; CI: 1.045–1.555; *p* -value = 0.02) and from 20.29% to 23.63% (PR: 1.164; CI: 1.004–1.350; *p* -value = 0.04) respectively. In the other hand, the prevalence in group 1 decreased from 20.91% to 16.97% (PR: 0.811; CI: 0.688–0.956; *p* -value = 0.01).

**Table 2:** Frequency of each group in the Robson Ten Group Classification compared before and during the pandemic

Group	Prepandemic: n (%)	Pandemic: n (%)	Prevalence ratio (confidence interval)	*p* -value
1	270 (20.91)	204 (16.97)	0.811 (0.688–0.956)	0.01
2	155 (12.01)	184 (15.31)	1.275 (1.045–1.555)	0.02
3	289 (22.39)	258 (21.46)	0.958 (0.827–1.112)	0.58
4	118 (9.14)	101 (8.40)	0.919 (0.713–1.185)	0.52
5	262 (20.29)	284 (23.63)	1.164 (1.004–1.350)	0.04
6	17 (1.32)	6 (0.50)	0.379 (0.150–0.958)	0.03
7	30 (2.32)	21 (1.75)	0.752 (0.433–1.306)	0.30
8	30 (2.32)	17 (1.41)	0.609 (0.337–1.10)	0.09
9	1 (0.08)	2 (0.17)	2.148 (0.195–23.66)	0.52
10	119 (9.22)	125 (10.40)	1.128 (0.889–1.432)	0.32
1 to 4	832 (64.45)	747 (62.15)	0.964 (0.901–1.024)	0.23
2 and 4	285 (23.71)	273 (21.15)	1.121 (0.969–1.300)	0.12
1 and 3	559 (43.33)	462 (38.44)	0.888 (0.807–0.967)	0.01

The overall CSR increased from 39.66% before the pandemic to 44.01% during it (PR: 1.110; CI: 1.011–1.217; *p* -value = 0.03). Data are presented in Table 3 . The CSR increased in almost every group, except groups 4 and 8. It presented a marked increase in group 5, in which the rate rose from 66.41% to 73.24% (PR: 1.103; CI: 0.987–1.232; *p* -value = 0.08). It also significantly increased among women undergoing induction of labor (groups 2 and 4), among whom the combined CSR increased from 32.92% to 47.02% (PR: 1.428; CI: 1.186–1.719; *p* -value < 0.01).

**Table 3:** Overall and per Robson Ten Group Classification rates of cesarean section compared before and during the pandemic

Group	Prepandemic: n (%)	Pandemic: n (%)	Prevalence ratio (confidence interval)	*p* -value
1	49 (18.15)	41 (20.10)	1.101 (0.783–1.608)	0.59
2	101 (65.16)	120 (65.22)	1.001 (0.856–1.170)	0.99
3	15 (5.19)	17 (6.59)	1.269 (0.647–2.490)	0.49
4	38 (32.20)	31 (30.69)	0.953 (0.643–1.412)	0.81
5	174 (66.41)	208 (73.24)	1.103 (0.987–1.232)	0.08
6	17 (100.00)	6 (100.00)	NS	NS
7	28 (93.33)	20 (95.24)	1.020 (0.891–1.168)	0.78
8	16 (94.12)	25 (83.33)	0.885 (0.725–1.080)	0.29
9	1 (100.00)	2 (100.00)	NS	NS
10	64 (53.78)	68 (54.40)	1.011 (0.803–1.275)	0.92
1 to 4	203 (24.40)	209 (27.98)	1.147 (0.971–1.354)	0.10
2 and 4	134 (32.92)	134 (47.02)	1.428 (1.186–1.719)	< 0.01
1 and 3	64 (11.45)	58 (12.55)	1.097 (0.786–1.530)	0.59
Total	512 (39.66)	529 (44.01)	1.110 (1.011–1.217)	0.03

To better understand the reasons behind the increase in the CSR, we observed the three major indications for CS in both periods, according to the medical charts. Before the pandemic, fetal distress (26.95%) was the leading indication, followed by repeated CS (15.63%) and maternal request (9.18%). During the pandemic, maternal request reached the first position (25.33%; PR: 2.759; CI: 2.025–3.759; *p* -value < 0.01), while fetal distress decreased its contribution (20.98%; PR: 0.778; CI: 0.626–0.968; *p* -value = 0.02). Table 4 summarize these findings.

**Table 4:** The three major indications for cesarean section before and during the pandemic

Indication	Prepandemic: n (%)	Pandemic: n (%)	Prevalence ratio (confidence interval)	*p* -value
Maternal request	47 (9.18)	134 (25.33)	2.759 (2.025–3.759)	< 0.01
Fetal distress	138 (26.95)	111 (20.98)	0.778 (0.626–0.968)	0.02
Repeated cesarean section	80 (15.63)	78 (14.74)	0.944 (0.708–1.258)	0.69

We observed that the frequency of CSs due to maternal request increased significantly among women undergoing induction of labor. In group 2, maternal request for CS increased from 11.80% to 36.70% (PR: 3.091; CI: 1.733–5.552; *p* -value < 0.01). It also increased in group 4 (from 10.50% to 33.33%; PR: 3.173; CI: 1.105–9.105; *p* -value = 0.021). Among women with at least 1 previous CS, who were included in group 5, the maternal request rose from 14.90% to 34.30% (PR: 2.297; CI: 1.543–3.431; *p* -value < 0.01).

## Discussion

During the pandemic, there was an overall increase in CSRs, mainly due to increased maternal request, in addition to an increase in the number of primiparous women admitted for induction of labor or elective CS (group 2), as well as a reduction in the admission of primiparous women in spontaneous labor (group 1).

Comparing data from our hospital with data from the Southeastern region of Brazil in a study 13 conducted from 2014 to 2016 regarding the distribution in the Robson groups, some differences were observed before the pandemic, especially in groups 1 (20.91% versus 14.4%) and 2 (12.01% versus 20.3%), which became more similar during the pandemic, when we noticed a decrease in group 1 (16.97%) and an increase in group 2 (15.31%).

A Brazilian study 14 among pregnant women reported that only 1/3 (27.6%) preferred CS at the beginning of antenatal care; however, 73.2% of those with a previous CS performed in the private health care system wanted a new cesarean, and fear of vaginal birth was the most mentioned factor for this choice, especially among primiparous women. On the other hand, most of the questioned obstetricians confirmed that they performed CSs through defensive medicine, concerned with the risk of judicialization. 15 These two issues, together with the lack of prior information about childbirth, can help explain the rate of cesarean deliveries in Brazil.

A study 16 comparing 3 months of the pandemic with the previous year showed an increase in nulliparous pregnant women (9% versus 12.5%) and pregnant women who arrived at the hospital in a more advanced stage of labor (26.8% versus 40%) during the pandemic. Another retrospective and comparative study 17 conducted in the United Kingdom, no significant differences were found in the frequency of cesarean births (31.2% versus 29.4%; *p* -value = 0.039). These results are different from our findings, since our data showed a decrease in the number of women admitted in spontaneous labor and an increase in CSs. This suggests that fear of exposure to COVID-19 and its potential risks may have delayed access to health care for women in labor; nevertheless, it could also have led to earlier requests for CS, prior to labor.

An important confounding factor in our results was the institution of a state law that guaranteed pregnant women the right to choose the mode of delivery, without the necessary explanation of the risks and benefits of the procedure during prenatal care. The impact of this law may have contributed, concurrently to the pandemic, to the increase in CSRs observed in the present work.

Important issues, such as the impossibility of having a companion, the length of stay for induction until delivery, reduction in medical and healthcare staff due to absences related to COVID-19 and even structural changes in the hospital may have led many pregnant women and doctors to choose CS. Continuous support during childbirth, by a person chosen by the woman, has significant benefits for the parturient and her children. 18 During the pandemic at our institution, not even patients without COVID had companions during childbirth.

The present study has several limitations, the retrospective data collection through medical chart reviews; however, we consider that this problem is partially mitigated, since all deliveries that occurred at the facility are audited weekly, and data on parity, mode of delivery, and the Robson Classification are reviewed, and divergences are prospectively corrected. In the other hand, we believe that the present is the first study that evaluated CSRs using the Robson's Classification during the COVID-19 pandemic in Brazil.

The hospital in which the present study was conducted was not a referral center for COVID-19 during pregnancy and universal screening was not implemented, with no data on the prevalence of the infection among women admitted for childbirth. However, it was a referral for COVID-19 among adults, and that supported many of the structural changes with impact on obstetric care, such as those aforementioned.

## Conclusion

Our results show that there was an increase in inductions among primiparous women and in the overall CSR during the analyzed period, with increased maternal requests for the procedure, especially among patients with a previous CS.
